# *Pseudomonas*-specific *16S rRNA* insect gut-microbiome profiling using next-generation sequencing

**DOI:** 10.1016/j.xpro.2022.101941

**Published:** 2022-12-16

**Authors:** Ayushi Gupta, Suresh Nair

**Affiliations:** 1 Plant-Insect Interaction Group, International Centre for Genetic Engineering and Biotechnology (ICGEB), Aruna Asaf Ali Marg, New Delhi 110067, India

**Keywords:** Genomics, Immunology, Microbiology, Molecular Biology, Sequencing

## Abstract

We present a detailed protocol for *Pseudomonas*-specific *16S rRNA* gut-microbiome profiling of brown planthopper (BPH) populations collected across changing climates and geographical locations using next-generation sequencing. We provide a technique for comparative analysis of *Pseudomonas* species structure and composition across BPH populations. Additionally, using qPCR we quantify the titers of *Pseudomonas* species in BPH. This protocol can be adopted for analyzing microbiome dynamics and monitoring populations of other pests, a crucial aspect for understanding their biodiversity, speciation, and adaptations.

For complete details on the use and execution of this protocol, please refer to Gupta et al. (2022).^1^

## Highlights

• *Pseudomonas*-specific *16S rRNA* gut-microbiome profiling of BPH populations
• Semiquantitative comparison of *Pseudomonas* titers in BPH populations
• Elucidation of the role of microbiome dynamics in facilitating insect adaptations
• Protocol can be adopted for understanding microbiome dynamics of other pest populations

## Abstract (editor highlights)

Publisher’s note: Undertaking any experimental protocol requires adherence to local institutional guidelines for laboratory safety and ethics.

## Graphical abstract

**Figure:**
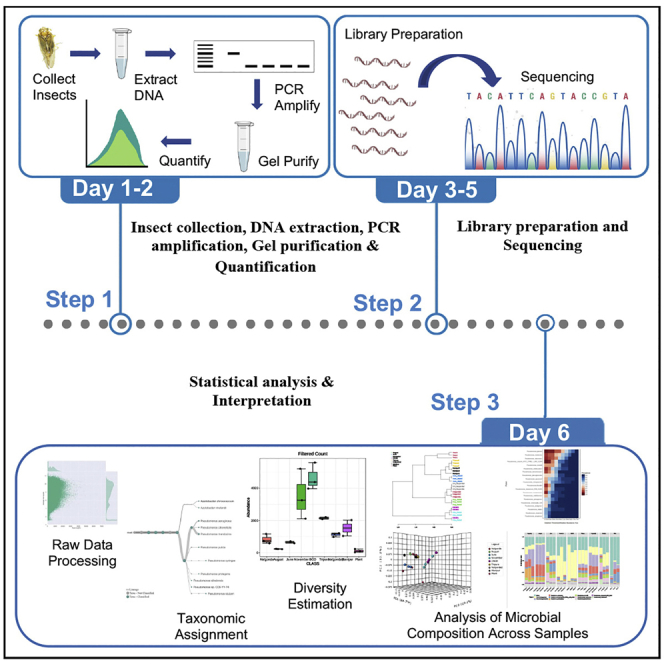

## Before you begin

The protocol below describes the specific steps for *Pseudomonas*-specific *16S rRNA* gut-microbiome profiling of the brown planthopper populations (BPH) collected from different geographical regions and across seasons. Additionally, this protocol can be successfully extended for screening and understanding the microbiome dynamics in the populations of other insect pests.

### Sample collection

Insects from geographically distant regions and thriving under different climatic and environmental conditions should be collected and preserved in absolute ethanol at −20°C until further use.

### Design primers

1. Design PCR primers specific for the reference gene (e.g., *β-Actin*) corresponding to the insect (BPH) genome being investigated.
  a. Input the gene sequence in MacVector (version 15.5) or any other primer design tool to design forward and reverse primers.
    i. Set the length of the PCR product between 100 to 200 bp. Avoid regions forming secondary structures and repeat motifs.
    ii. Set the primer length between 20 and 24 bp.
    iii. GC content is set between 45 and 60%, and T_m_ is kept between 60°C and 65°C.
    iv. Check the sequences of forward and reverse primers and ensure that there is no 3′ complementarity (i.e., avoid primer-dimer formation).

***Note:*** When choosing primers, check for self-dimer and hetero-dimer scores. The ΔG value for these scores should be weaker (more positive) than -9.0 kcal/mole. While, any commercially available software can be used for primer design, we used the MacVector suite of sequence analysis programmes for designing suitable primers. Second, perform an ePCR before finalizing your primer pair against the non-redundant nucleotide database (if available) to ensure specificity. If ePCR indicated the generation of PCR products from non-specific regions, redesign the primers. This can be carried out using the BLAST tool hosted by NCBI (https://www.ncbi.nlm.nih.gov/tools/primer-blast/). Alternatively, the specificity of primer pair can be checked by performing a BLAST using an inbuilt tool provided in MacVector.

## Key resources table

**Table:** 

REAGENT or RESOURCE	SOURCE	IDENTIFIER
**Biological samples**		

Cells, tissues, whole body	Insects	N/A

**Chemicals, peptides, and recombinant proteins**		

Deoxynucleotide (dNTPs) Solution Mix	New England BioLabs, USA	Cat#N0446S
Taq DNA polymerase	Bangalore GeNei, India	Cat#0601600051730
Imidacloprid (Confidor 17.80% SL)	Bayer AG, Germany	N/A
Gene ruler 1Kb DNA ladder	Thermo Scientific, USA	Cat#SM0311

**Critical commercial assays**		

GF-1 Tissue DNA Extraction kit	Vivantis, Malaysia	Cat#GF-TD-050
GeneJET Plant Genomic DNA Purification kit	Thermo Scientific, USA	Cat#K0791
Qubit dsDNA BR Assay kit	Invitrogen, USA	Cat#Q32853
GF-1 AmbiClean kit (Gel & PCR)	Vivantis, Malaysia	Cat#GF-GC-100
NEBNext Ultra II DNA Library Prep Kit for Illumina	New England BioLabs, USA	Cat#E7645L
PCR Barcoding Expansion 1–96 kit	Oxford Nanopore Technology, UK	Cat#EXP-PBC096
Ligation sequencing kit	Oxford Nanopore Technology, UK	Cat#SQK-LSK109

**Deposited data**		

Sequence Read Archive (SRA) files	NCBI, https://www.ncbi.nlm.nih.gov/	GenBank: PRJNA733325

**Oligonucleotides**		

Pseudo-S2-F	5′-GACGGGTGAGTAATGCCTA-3′	Spilker et al.^2^
Pseudo-S2-R	5′-CACTGGTGTTCCTTCCTATA-3′	Spilker et al.^2^
ACT-mod F	5′-TGCGTGACATCAAGGAGAAGCTG-3′	Gupta and Nair^3^
ACT-mod R	5′-GTACCACCGGACAGGACAGT-3′	Gupta and Nair^3^

**Software and algorithms**		

Albacore v2.3.1.	Oxford Nanopore Technology, UK	N/A
MinKNOW 2.1 v18.05.5	Oxford Nanopore Technology, UK	N/A
ranacapa R package	Kandlikar et al.^4^	N/A
EPI2ME Agent software	Oxford Nanopore Technology, UK	N/A
MicrobiomeAnalyst software	https://www.microbiomeanalyst.ca/	N/A
Phyloseq R package	McMurdie and Holmes^5^	N/A
R package microbiome	http://microbiome.github.io	N/A
Image Lab software v6.0.1	Bio-Rad Laboratories, USA	N/A
MacVector v.15.5	https://macvector.com/	N/A
SPSS Statistics v.22.0	IBM, USA	N/A

**Other**		

Qubit 4.0 fluorometer	Invitrogen, USA	Cat#Q33226
Gel Documentation System - Alpha Imager HP	Protein Simple, USA	Cat#921382400
NanoVue Plus Spectrophotometer	GE Healthcare, UK	Cat#28-9232-15
GridION X5	Oxford Nanopore Technology, UK	Cat#GRD-X5B003
SpotON flow cell R9.4	Oxford Nanopore Technology, UK	Cat#FLO-MIN106D
Veriti 96 well thermal cycler	Applied Biosystems, USA	Cat#4375786

## Materials and equipment

**Table:** TBE (Tris-Borate-EDTA) buffer

Reagent	Final concentration	Amount
Tris Base	10 ×	108 g
Boric Acid	10 ×	55 g
EDTA (0.5 M; pH 8.0)	10 ×	40 mL
ddH_2_O	N/A	900 mL
**Total**	**N/A**	1 L

***Note:*** Dilute stock solution 10:1 to make a 1× working solution.

## Step-by-step method details

### Sample collection

1. Insect populations.
  a. Collect insect populations to be screened for the presence of *Pseudomonas* from different geographical regions and climatic zones.
    ***Optional:*** A lab-reared population maintained under suitable growth conditions (i.e., unexposed to stress) can be used as a control.
  b. Preserve all collected insects in absolute ethanol (99.9%) and store at −20°C till further use.
2. Plant samples.
  a. In the case of phytophagous insects, dissect regions representing the feeding site, from both infested and uninfested plants grown in sterile vermiculite, to determine the influence of the host plant on the insect’s gut microbiome.
  b. Preserve the dissected tissues in absolute ethanol (99.9%) and store at −20°C till further processing.

### Total DNA extraction from rice and BPH and quality check

**Timing: 5–6 h (for step 3)**

This step describes the protocol for extracting the genomic DNA from insects and plant tissues, for *Pseudomonas*-specific microbiome profiling.

**Figure 1:**
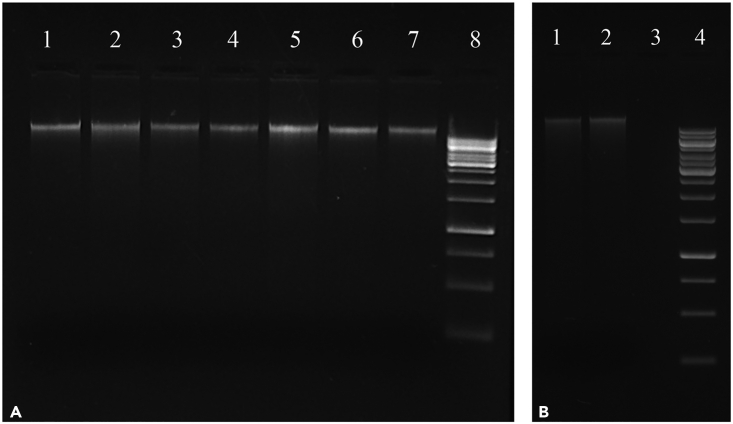
Representative 0.8% agarose gel electrophoresis image of the extracted genomic DNAs (A). Insect samples (Lanes 1–7: DNA isolated from different BPH individuals; Lane 8: 1 Kb DNA ladder). (B) Plant samples (Lanes 1–2: DNA isolated from different rice seedlings; Lane 3: Blank; Lane 4: 1 Kb DNA ladder).

3. Isolate the total DNA from individual insects (∼3 mg dry weight) of each population using the GF-1 tissue DNA extraction kit (Vivantis, Malaysia) following the steps mentioned below:
  ***Note:*** It is advisable to isolate DNA from not more than 20 individuals at a time to avoid time lag and subsequent DNA degradation.
  a. Take out the insects (stored at −20°C in absolute alcohol) on a fresh Petri plate, de-wing using a sterile blade, and transfer to a fresh 1.5 mL Eppendorf tube.
  b. Grind the insect in liquid nitrogen and homogenize in 250 μL of Tissue lysis (TL) buffer (provided in the kit) using a sterile micro pestle.
    ***Note:*** This should be performed quickly to avoid DNA degradation.
  c. Briefly vortex the tubes and add 3 μL of RNase A (10 mg/mL, provided in the kit), seal with Parafilm, and incubate at 37°C for 30 min in a water bath.
  d. After RNase treatment, add 20 μL of proteinase K (20 mg/mL) and 12 μL of lysis enhancer to the tubes, invert gently for thorough mixing of solutions, and incubate at 65°C for 2–3 h until the tissue is completely dissolved.
  e. Add two volumes (∼600 μL) of Tissue binding (TB) buffer (provided with the kit). Mix thoroughly and incubate at 65°C for 10 min.
  f. Add 200 μL of absolute ethanol. Mix immediately and thoroughly by quick vortexing to obtain a homogenous solution.
  g. Transfer ∼600 μL of sample into the column assembled in a clean collection tube.
  h. Centrifuge at 7000 g for 1 min. Discard the flow through.
  i. Repeat the above steps (steps g, h) for the remaining samples.
  j. Wash the column with 600 μL of wash buffer and centrifuge at 7000 g for 1 min.
  k. Discard the flow through.
  l. Subject the column to a short dry spin to remove all traces of ethanol. Discard the flow through.
  m. Next, place the column on a fresh 1.5 mL Eppendorf tube, and add 36 μL of pre-warmed elution buffer (EB; provided with the kit) to the center of the column. Let it stand for 5 min at room temperature (28°C–37°C).
  n. Spin the tubes at 19,000 g for 1 min.
  o. The eluate contains purified DNA.
  p. DNA integrity, purity and concentration were checked using gel electrophoresis^6^ and by measuring absorbance on NanoDrop spectrophotometer (Thermo Scientific, USA). The absorbance ratio 260/280 when ≥1.8, is indicative of a pure DNA sample. And the absorbance ratio 260/230, when <1.8, indicates contamination by organic compounds and/or chaotropic agents.
    ***Note:*** The approximate amount of DNA obtained from a single adult BPH usually ranges from 1.5 - 2 μg. Extracted DNA is to be kept on ice and stored at −20°C till further use.

4. Isolate the total DNA from plant samples using the GeneJET Plant Genomic DNA Purification mini kit (Thermo Scientific, USA) as per the protocol mentioned below:
  ***Alternatives:*** Any plant genomic DNA isolation kit can be used here to extract the DNA.
  a. Grind the plant tissue (100 mg) in liquid nitrogen.
  b. Immediately transfer the tissue powder to 1.5 mL Eppendorf tube containing 350 μL of lysis buffer A (supplied with the kit).
    ***Note:*** This should be performed quickly to avoid DNA degradation.
  c. Vortex for 15 s and mix thoroughly.
  d. Add 50 μL of lysis buffer B and RNase A (provided with the kit).
  e. Incubate the sample for 10 min at 65°C (preferable in a water bath) with occasional vortexing.
  f. Add 130 μL of precipitation solution and mix by inverting the tube 1–3 times.
  g. Incubate on ice for 5 min.
  h. Centrifuge at 22,000 g for 5 min.
  i. Collect the supernatant and transfer it to a clean Eppendorf tube.
  j. Add 400 μL of Plant gDNA Binding solution and 400 μL of 96% ethanol. Mix well.
  k. Transfer half of the prepared mixture to the spin column (provided with the kit) and centrifuge at 7000 g for 1 min.
  l. Discard the flow through and apply the remaining mixture onto the same column.
  m. Centrifuge for 1 min at 7000 g.
  n. Add 500 μL of Wash Buffer I (reconstituted in absolute ethanol) to the column and centrifuge for 1 min at 11,500 g.
  o. Discard the flow-through and place the column back into the collection tube.
  p. Add 500 μL of Wash Buffer II to the column and centrifuge for 3 min at 22,000 g.
  q. Discard the flow-through and place the column back into the collection tube.
  r. Perform dry centrifugation at full speed for 1 min to remove the traces of residual ethanol.
  s. For elution, use 100 μL of pre-warmed EB applied at the center of the column and incubate for 5 min at room temperature (28°C–37°C). Place the column on a fresh Eppendorf tube.
    ***Note:*** Using pre-warmed elution buffer at the DNA elution step results in higher yields.
  t. Centrifuge for 1 min at 11,200 g.
  u. The eluate contains purified DNA.
    ***Note:*** Extracted DNA is to be kept on ice and stored at −20°C till further use.

5. Quantify the extracted genomic DNA (from insect and plant tissues) on the Qubit 4.0 fluorometer (Invitrogen, USA) as per the instructions mentioned below.
  a. Set up the required number of Qubit tubes for samples.
  b. Label the tube lids.
    **CRITICAL:** Do not label the side of the tube as this could interfere with the sample reading.
  c. Prepare the Qubit working solution by diluting the Reagent in a 1:200 ratio in Buffer.
  d. Add 198 μL of working solution to each sample tube.
  e. Add 2 μL of each sample to the appropriate tube, then mix by vortexing. The final volume should be 200 μL.
  f. Incubate all the tubes at room temperature (28°C–37°C) for 2 min.
  g. Proceed to quantifying samples on Qubit fluorometer.
  h. On the home screen of the Qubit, press dsDNA, then select dsDNA: High Sensitivity or dsDNA: Broad Range depending on the kit being used.
    ***Note:*** We used dsDNA: Broad Range kit for quantification.
  i. Press Read Samples to proceed.
  j. In the Sample Volume screen, select the volume of sample added to the tube and the output concentration units (i.e., ng/μL).
  k. Insert the first sample tube into the sample chamber, close the lid, and then press Read Tube.
  l. The software displays the results of the sample. The top value (in large font) is the concentration of the original sample. The bottom value is the dilution concentration. Record the concentration of the original sample, remove the tube, and repeat readings and results recording for each additional sample.
6. Assess the quality of the DNA by gel electrophoresis (Figure 1, using 0.8% TBE agarose gel^6^). The concentration of DNA typically ranges from 20–60 ng/μL and 50–150 ng/μL for insect and plant samples, respectively. However, these values may change depending on the amount of starting material.

### Screening insect populations for the presence of *Pseudomonas*

7. The *Pseudomonas-*specific V3-V4 hypervariable region of *16S rRNA* (for species-level identification) is to be PCR amplified using insect DNA as a template, and primer pair Pseudo-S2-F 5′-GACGGGTGAGTAATGCCTA-3′ and Pseudo-S2-R 5′-CACTGGTGTTCCTTCCTATA-3'.

**Table:** PCR reaction master mix

Reagent	Amount
DNA template (25 ng)	1 μL
DNA Polymerase (3 U/μL)	0.2 μL
Pseudo S2-F (150 ng/μL)	0.4 μL
Pseudo S2-R (150 ng/μL)	0.4 μL
Buffer (10×)	2 μL
dNTPs (1.5 mM)	3.2 μL
MilliQ H_2_O	12.8 μL

**Table:** PCR cycling conditions

Steps	Temperature	Time	Cycles
Initial Denaturation	95°C	5 min	1
Denaturation	95°C	30 s	25 cycles
Annealing	58°C	1 min	
Extension	72°C	45 s	
Final extension	72°C	5 min	1
Hold	4°C	till samples are removed

**Figure 2:**
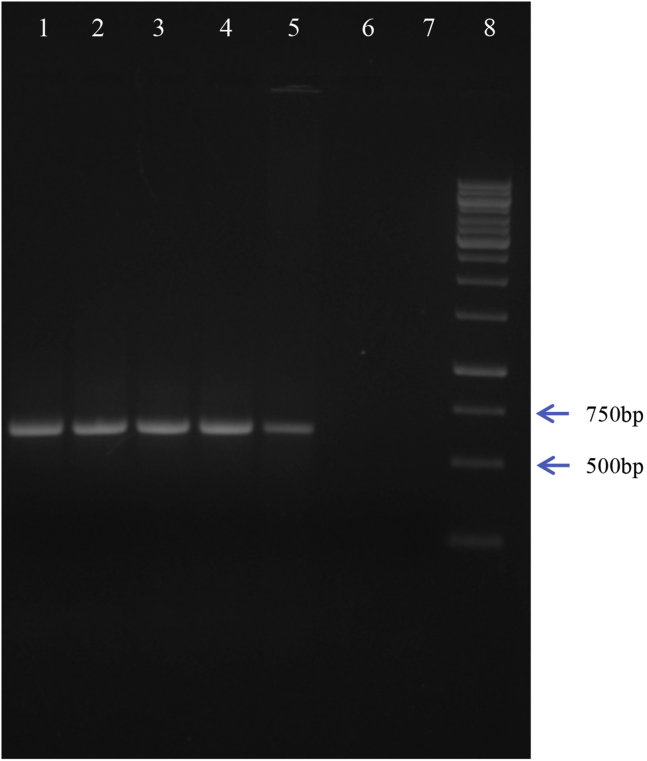
0.8% agarose gel showing PCR amplified 617 bp fragment corresponding to the *Pseudomonas-*specific *16S rRNA* Lanes 1–5: BPH samples, Lanes 6–7: Blank, Lane 8: 1Kb DNA ladder. Arrows on the right indicate the molecular weights of the corresponding fragments of the 1Kb ladder.

8. The PCR amplified products should be separated on 0.8% TBE agarose gel (Figure 2).

### Purification of PCR-amplified *16S rRNA* fragments

**Timing: 1–2 h (for step 9)**

This step describes the protocol for purifying PCR-amplified fragments for sequencing.

9. Purify the PCR-amplified fragments (618 bp; obtained from the insect populations harboring *Pseudomonas*) using GF-1 AmbiClean (Gel & PCR) kit (Vivantis, Malaysia).
  ***Note:*** Any DNA elution kit can be used here to purify and extract PCR amplified fragments from the agarose gel.
  a. Visualize and identify the appropriate PCR-amplified fragment on the gel under a UV transilluminator. Using a sterile blade, excise the band of interest (618 bp).
    **CRITICAL:** Exposure of gel containing the PCR amplified product to UV should be kept to a minimum.
  b. Transfer the gel piece to a pre-weighed Eppendorf tube.
  c. Determine the net weight of gel slice, and add equivalent (1 vol) volume of DB buffer (i.e., 100 μL DB buffer should be added per 0.1 g gel).
  d. Incubate the tubes at 50°C with occasional mixing until the gel slice completely dissolves.
  e. Add 1 vol absolute ethanol and quickly vortex for 5 s.
  f. Load the mixture onto the DNA binding column provided with the kit and centrifuge at 13,500 g for 1 min.
  g. Discard the flow through.
  h. Add 650 μL of wash buffer and centrifuge at 13,500 g for 1 min.
  i. Discard the flow through.
  j. Subject the column to a short dry spin to eliminate all the traces of ethanol.
  k. Discard the flow through.
  l. Place the column on a fresh Eppendorf tube, and add 36 μL of elution buffer (EB) to the center of the column. Let it stand for 5 min at room temperature (28°C–37°C).
  m. Spin the tube at 13,500 g for 1 min. The eluate contains the purified PCR DNA.

Quantify the eluted product using the Qubit dsDNA Assay BR kit on Qubit 4.0 fluorometer (Invitrogen, USA). The expected concentration of the purified PCR product ranges from 10–15 ng/μL. Usually, DNA recovery, using the above-mentioned protocol, is >80%. Sequence the isolated fragments using the Oxford Nanopore Technology (ONT, UK).

***Note:*** Nanopore sequencing technology enables direct, real-time analysis of long DNA fragments. Hence, we prefer Nanopore sequencing over conventionally used Illumina sequencing platform. Alternatively, PacBio sequencing platform can also be used.

### Construction of *Pseudomonas*-specific *16S rRNA* library and sequencing

**Timing: 1–2 days (for step 10)**

Library preparation is a critical step towards obtaining good quality sequencing reads, hence must be carried out carefully following the manufacturer’s instructions.

10. NEBNext End Prep.

For library preparation, approximately 300 ng of the eluted PCR product, representing each amplicon, must be end-repaired using the NEBnext ultra II end repair kit (New England Biolabs, MA, USA). Clean up the reaction using with 1× AmPure beads (Beckmann Coulter, USA).

11. Barcoding Adapter ligation.

Perform the barcoding adapter ligation (BCA) using the NEB blunt/TA ligase (New England Biolabs, MA, USA) following manufacturer’s instructions and clean the reaction with 1× AmPure beads. Quantify the barcoding adapter-ligated DNA using a Qubit 4.0 fluorometer (Invitrogen, USA).

12. PCR Barcoding.

Attach barcodes to the adapter-ligated amplicons through PCR using the corresponding barcode primers and LongAmp Taq polymerase (LongAmp Taq 2×, New England Biolabs, MA, USA) as per the manufacturer’s instructions. Clean-up the reaction mixture with 1.6× AmPure beads (Beckmann-Coulter, USA).

13. Pooling.

Pool the purified barcoded amplicons in equal proportions from all the barcoded samples.

14. End repairing of pooled DNA.

Carry out the end-repairing step with the pooled sample using the NEBnext ultra II end repair kit and clean up with 1× AmPure beads.

15. Adapter ligation and sequencing.

Perform 1D adapter a ligation for the end-repaired amplicons using NEB blunt/TA ligase (New England Biolabs, MA, USA) and clean up using 0.4× Ampure beads (Beckmann Coulter, USA). Elute the library in 16 μL of elution buffer for nanopore sequencing.

***Note:*** We used EXP-PBC096 and SQK-LSK108 kits procured from ONT (Oxford Nanopore Technology, UK) for barcoding and library preparation. Sequencing was performed on GridION X5 (Oxford Nanopore Technologies, Oxford, UK) using the SpotON flow cell (R9.4) in a 48 h sequencing protocol on MinKNOW 2.1 v18.05.5.

***Alternatives:*** Commercially available sequencing services can be availed for library construction and sequencing.

### Generation of raw data, base-calling, and de-multiplexing

16. Nanopore generates raw sequencing reads in the *fast5* format, which are then subjected to base calling and de-multiplexing using Albacore v2.3.1 (ONT, UK).

***Note:*** Base calling converts the data to *fastq* format which is the preferred format for downstream processing.

### Taxonomic assignment and species identification

**Timing: 30 min (for step 17)**

The following describes the steps required for the identification and classification of *Pseudomonas* species present across insect populations.

17. Upload the base-called read files to the EPI2ME platform via EPI2ME Agent software (ONT, UK).
18. Perform the quality assessment and microbial classification to identify different *Pseudomonas* species present across samples using the Fastq 16S workflow.
  a. First, filter the reads by the quality and then subject to adapter trimming and barcode detection.
  b. Carry out the taxonomy assignment using BLAST in conjunction with the NCBI database while keeping minimum horizontal coverage of 30% and a minimum accuracy of 77% (ONT, UK).

***Note:*** Pre-configured alignment parameters such as identity and coverage of sequences should be used for analysis.

**CRITICAL:** Same parameters must be used to analyse all the samples included in the study.

### Diversity estimation

**Timing: 2–3 h (for step 19)**

Download the taxonomic assignment results as .csv files for each sample for performing downstream analyses such as diversity and taxonomic differential abundance estimation.

**Figure 3:**
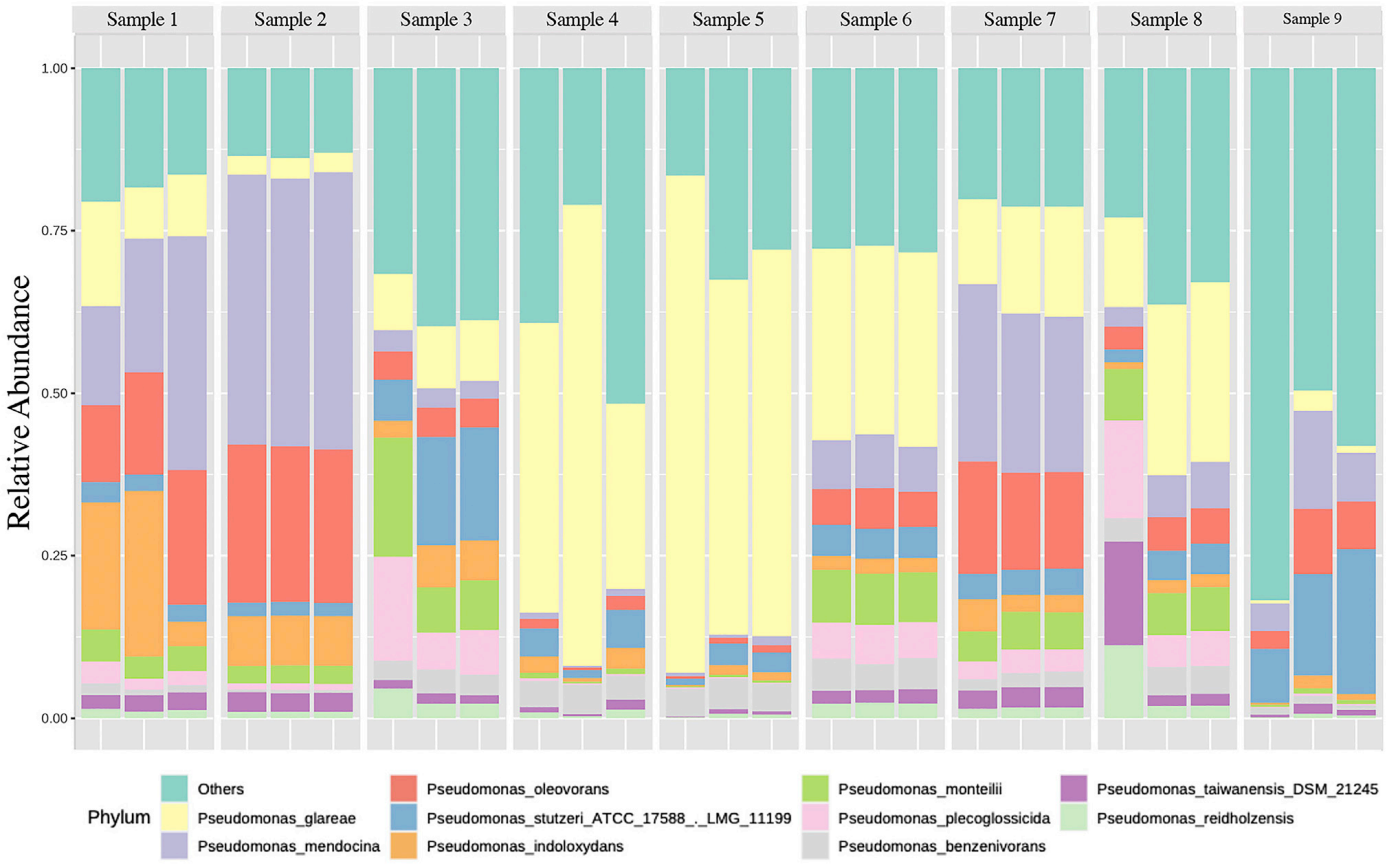
A representative stacked bar plot depicting the variation in *Pseudomonas* species composition, their relative abundance and diversity between samples

***Note:*** The top 10 taxa present in each sample can be plotted while the ones with very low read counts can be merged for better visualization of significant taxonomic patterns.

19. Determination of taxonomic composition.
  a. Perform the rarefaction curve analysis using the modified function ‘ggrare’ (ranacapa R package^4^) to determine whether sequencing depth was sufficient to discover all the *Pseudomonas* species present in the samples.
  b. To identify and remove taxa that are unlikely to be of further use while modeling the data, filter out the species having very few counts based on their abundance level (minimum counts) across samples (prevalence). The purpose of the data filtering is to identify and remove features that are unlikely to be of use when modeling the data.
  c. Post filtration, scale and normalize the data for all the samples to get rid of uneven sequencing depths using the MicrobiomeAnalyst software with default parameters (https://www.microbiomeanalyst.ca/). By default, features having zero counts across all the samples or those that appear only in one sample are removed from further analysis. Subsequently, data rarefaction is performed, followed by total sum normalization based on the total sum scaling method.
  d. The community’s taxonomic composition and relative abundance can be visualized across samples using a stacked bar plot generated by MicrobiomeAnalyst (Figure 3).

20. Diversity estimation.
  Perform the diversity estimation using the ‘phyloseq’ package (R package^5^ version 1.19).
  a. Determine the alpha-diversity to assess the species richness and diversity present within samples based on Chao1, Shannon and Simpson indices.^7^^,^^8^^,^^9^
  b. Calculate beta-diversity index using the Jenson-Shannon divergence index followed by the Principal Coordinate Analysis (PCoA).
  c. Based on beta-diversity indices compare the taxonomic diversity across samples. This can be visualized as a 3D plot, with each point representing the entire microbiome of a single sample.
    ***Note:*** Samples with similar microbiome composition will be clustered together.

21. Identification of core microbiome.
  a. Identify the *Pseudomonas* species that remain unchanged in their composition across samples based on their prevalence and relative abundance in insect populations using the ‘core’ function in R package ‘microbiome’.
  b. Generate the heat map using MicrobiomeAnalyst software.
22. Perform the hierarchical clustering analysis for insect populations based on the overall variation in species composition and abundance, using the ‘hclust’ function in R package ‘stat’.

***Note:*** For this, use the Jaccard index to measure the distance between samples, and Ward’s linkage algorithm for clustering.

23. Generate dendrogram for better visualization of the results obtained upon clustering analysis in MicrobiomeAnalyst.

### Semi-quantitative PCR (qPCR) for pesticide-exposed and -unexposed insects

24. Estimate the LD_50_/LC_50_ (i.e., lethal dose/concentration that kills 50% of the test population) imidacloprid (Confidor 17.80% SL; Bayer AG, Germany) for the control insect populations (unexposed to stress) to ascertain their pesticide resistance/tolerance status.
  a. LD_50_ estimation.
    i. Anesthetize the control insects (adults) on ice for 3–4 min.
    ii. Apply the pesticide solution (0.5 μL/insect) onto anesthetized individuals using a micropipette and allow it to dry for 10–15 min.
    iii. Test different concentrations of imidacloprid (active ingredient (a.i.) 0.5 ng–4.0 ng).
    iv. Release the imidacloprid treated insects onto susceptible host plants.
    v. Count the live insects 24 and 48 h after treatment.
  b. LC_50_ estimation.
    i. Spray the susceptible host plant with different concentrations of pesticide (imidacloprid) solution (ranging from 0.02%–0.5% a.i.) and let it dry for ∼30 min.
    ii. Release the control insects onto the imidacloprid-treated plants.
    iii. Count the live insects 24 and 48 h after release.
25. Perform probit analysis for LD_50_/LC_50_ data using SPSS Statistics v. 22.0.

***Note:*** Both LD_50_/LC_50_ experiments must be carried out in triplicates with at least 10 insects per replicate and for each concentration.

26. Generate a pesticide-resistant insect population in the laboratory.
  a. A pesticide-resistant BPH population can be generated in the laboratory by periodically exposing the control insect population (∼50; adults) to imidacloprid.
  b. Initially, for the first two generations, the control insects (∼50; adults) must be exposed to LC_40_ imidacloprid solution, and the concentration can be gradually increased to LC_60_ imidacloprid.
  c. Spray the pesticide solution twice a week, using a spray bottle.
  d. Collect the imidacloprid-exposed individuals at different time points (generations).

***Note:*** Although we have analysed the influence of imidacloprid on *Pseudomonas* titers in BPH, this protocol can be successfully implemented to estimate the impact of other pesticides on insect populations belonging to other taxa.

27. Extract the total genomic DNA following the above-mentioned protocol from individual insects (adults) reared under imidacloprid stress.

***Note:*** Insects of different generations can be screened for pesticide-induced alteration in the *Pseudomonas* titers, while keeping lab-cultured insects as the experimental control.

28. Estimate *Pseudomonas* titers in insect populations, post-exposure to the pesticide, using semi-qPCR.
  a. Compare the titers of *Pseudomonas* in the pesticide-exposed and -unexposed individuals.
  b. *Pseudomonas*-specific primers Pseudo S2-F and Pseudo S2-R were used to amplify *Pseudomonas* (sequence details as mentioned before).
  c. Use the primers synthesized against the reference gene for normalization.
    ***Note:*** We have used the BPH Actin gene (Accession Number: KU196668.1) as the internal control for normalization. The primer pair used for *Actin* was ACT-mod F 5′-TGCGTGACATCAAGGAGAAGCTG-3 and ACT-mod R ‘5’-GTACCACCGGACAGGACAGT-3 and PCR conditions were as follows:
    **Table:** PCR reaction master mix

Reagent	Amount
DNA template (25 ng)	1 μL
DNA Polymerase (3 U/μL)	0.2 μL
ACT-mod F (150 ng/μL)	0.4 μL
ACT-mod R (150 ng/μL)	0.4 μL
Buffer (10×)	2 μL
dNTPs (1.5 mM)	3.2 μL
MilliQ H_2_O	12.8 μL
    **Table:** PCR cycling conditions

Steps	Temperature	Time	Cycles
Initial Denaturation	95°C	5 min	1
Denaturation	95°C	30 s	25 cycles
Annealing	58°C	30 s	
Extension	72°C	45 s	
Final extension	72°C	5 min	1
Hold	4°C	till samples are removed

    **CRITICAL:** Lesser number of cycles ensures that measurements of PCR products are made at the exponential phase of the PCR amplifications.
  d. Run the PCR products on 1% agarose gel and photograph using a gel documentation system (Alpha Imager, Cell Biosciences, UK).
  e. Quantify PCR yields based on the intensity of the band obtained for *Pseudomonas,* and normalize the reaction using the intensity of the PCR amplification of the reference (*Actin*) gene product from each sample.
  f. Measure the relative intensities for each fragment using the Image Lab software 6.0.1 (Bio-Rad Laboratories, USA).
    ***Note:*** Annealing temperature will depend on the primers designed against the reference gene. We recommend using a Tm calculator provided by the primer vendor or the thermal cycler’s gradient feature to find the suitable annealing temperature for the primer pair being used.

## Expected outcomes

**Figure 4:**
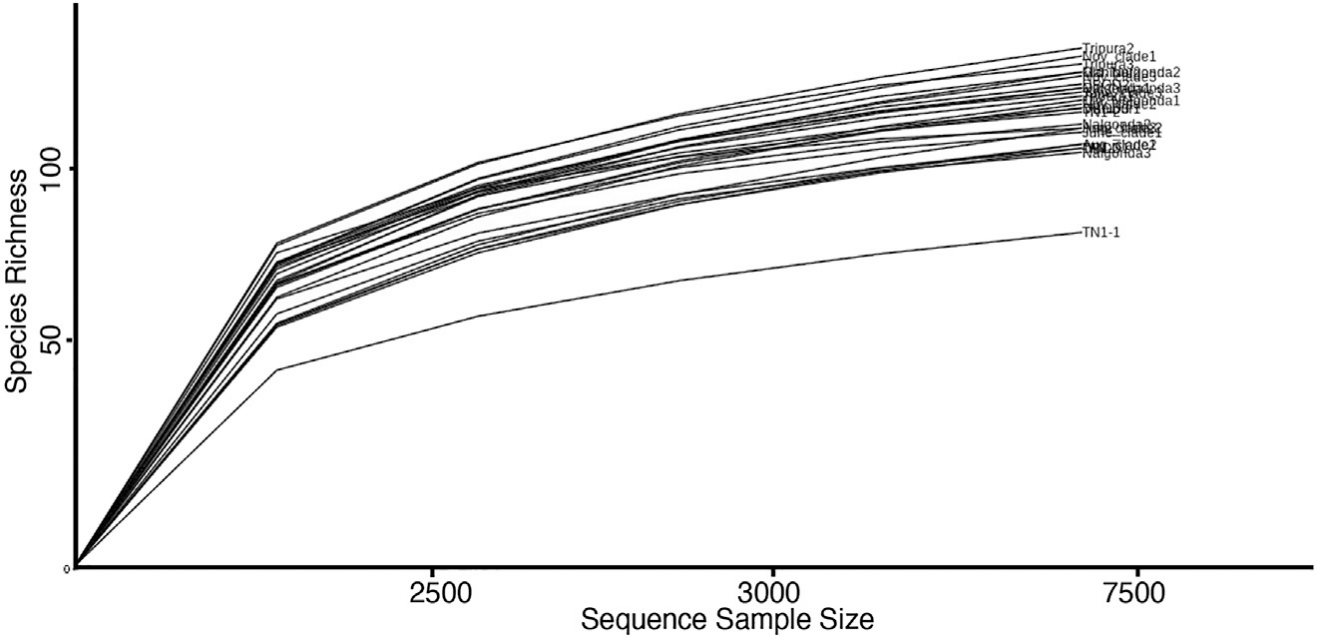
Saturation of rarefaction curves indicative of good sequencing depth and coverage

PCR amplification of insect genomic DNAs using primers specific for the hypervariable V3-V4 region of *Pseudomonas 16S rRNA* would yield a 618 bp fragment from insects harboring *Pseudomonas*. Nanopore sequencing libraries constructed from PCR-amplified *16S rRNA* fragment (618 bp) is expected to generate high-quality reads with a minimum of 100× coverage. Of the total reads obtained, >90% reads can be classified up to the species level with >85% accuracy on the EPI2ME platform. The rarefaction curves must plateau (Figure 4) indicating good sequencing depth and coverage. This is usually achieved by generating higher number of sequencing reads (i.e., >50,000) per sample. However, in cases where higher sequencing reads are not possible, rarefaction curves could be used as an indicator of having achieved adequate sequencing depth. And in addition, it also confirms whether a specific sample has been sufficiently sequenced to represent its identity and whether obtained sequences are suitable for subsequent analyses. Based on *Pseudomonas* species composition, diversity, and relative abundance, insect populations can be differentiated and grouped into discrete clusters. Further, depending on the physiological capacity and roles of individual species of *Pseudomonas* present across populations their involvement in facilitating insect survival and adaptations can be estimated. For instance, alterations observed in *Pseudomonas* titers after exposure to pesticides can be linked to its involvement in pesticide detoxification and resistance.^1^

## Quantification and statistical analysis

1. The statistical significance of the clustering pattern in the 3D plot (generated for beta-diversity estimations) should be evaluated using Permutational ANOVA (PERMANOVA^10^) with a p-value cut-off ≤ 0.001.
2. Identify the differentially abundant *Pseudomonas* species across insect populations using the DESeq2 statistical method^11^ for variance estimation with the adjusted p-value cut-off ≤0.05.
3. Perform the univariate analysis to determine the top 50 species having significant variation in abundance between samples.
4. Additionally, use a non-parametric factorial Kruskal-Wallis (KW) sum-rank test^12^ to identify species with significant differential abundance, followed by Linear Discriminant Analysis (LDA) to calculate the effect size of each differentially abundant species (p-value cut-off adjusted to 0.05).

***Note:*** All statistical analyses were carried out using r scripts integrated in MicrobiomeAnalyst.

## Limitations

The copy number of the amplicon is of vital importance in such studies; therefore, it is important to maintain good quality control of pipetting and template DNA used for PCR. Experimental deviations can lead to misinterpretation of the results obtained.

Further, while this protocol is a reliable, efficient and quick estimate of microbiome dynamics across populations, it is well-established that microbial community dynamics within insects fluctuate with changing external environments. And variation observed in the microbial composition and abundance (*viz*. *Pseudomonas* sp.) across insect (or any other host) populations is an outcome of remarkably complex interplay between insect physiology and the microbial community, which likely confers a rapid stress resistance/tolerance capacity to the host. However, as microbiome-host interplay is more complex, involving several other parameters other than microbial community dynamics, therefore, a more detailed exploration (other than microbiome profiling based on *Pseudomonas*) is necessary to fully understand and determine how microbial community dynamics regulate the insect’s responses to stress.

## Troubleshooting

### Problem 1

Ideally, the housekeeping gene and the 16S rRNA gene products should be amplified under the same PCR conditions. Sometimes, the housekeeping gene and 16S rRNA gene product do not amplify under identical PCR conditions.

### Potential solution

Preferably design additional primer pair sets for the housekeeping gene such that a suitable primer pair is identified and made available for rest of the experimentation.

### Problem 2

Due to low incidence of microbes, at times, the insect DNA may not amplify the 16S rRNA gene fragment or the amplification could be poor.

### Potential solution

In such cases where it is important to quantify the amplification product, increase the number of PCR cycles. Carry out several PCR trials to finalize the number of cycles before the final run.

***Note:*** Finally, all samples must be amplified at same PCR conditions.

### Problem 3

Experimental errors during quantification of 16S rRNA gene products.

### Potential solution

For the quantitation of 16S rRNA gene products and for studying differential amplification across several test samples using image analysis, it is important to ensure that the 16S rRNA gene products and their respective internal control products are run on the same agarose gel or run same samples across gels to normalize for gel-to-gel variations in band intensities.

### Problem 4

The precise quantitation of the gel-purified fragment for downstream processing is important specially for library construction.

### Potential solution

To avoid inconsistences in quantitation of PCR products based on spectroscopy quantitation, quantitation is best done using a fluorometric method.

### Problem 5

Low species level classification (i.e., >10% of the reads remains unclassified).

### Potential solution

This problem often arises due to lack of requisite sequence information in the database. Hence, to increase the number sequences that are classifiable to species level, carry out the analysis using different databases and also a few days after the first analysis with a more recent database.

***Note:*** EPI2ME resources are updated regularly.

## Resource availability

### Lead contact

Further information and requests for resources, reagents and strains should be directed to and will be fulfilled by the lead contact, Suresh Nair (suresh@icgeb.res.in).

### Materials availability

This study did not generate new unique reagents. Insect and plant samples used in this study are available from the lead contact with a completed Materials Transfer Agreement.

## Data Availability

NGS data have been deposited at NCBI as Sequence Read Archive (SRA) files and are publicly available as of the date of publication. Accession number is listed in the key resources table. This paper does not report original code.

## References

[1] Gupta A., Sinha D.K., Nair S. (2022). Shifts in *Pseudomonas* species diversity influence adaptation of brown planthopper to changing climates and geographical locations. iScience.

[2] Spilker T., Coenye T., Vandamme P., LiPuma J.J. (2004). PCR-based assay for differentiation of Pseudomonas aeruginosa from other Pseudomonas species recovered from cystic fibrosis patients. J. Clin. Microbiol..

[3] Gupta A., Nair S. (2022). Heritable epigenomic modifications influence stress resilience and rapid adaptations in the brown planthopper (*Nilaparvata lugens*). Int. J. Mol. Sci..

[4] Kandlikar G.S., Gold Z.J., Cowen M.C., Meyer R.S., Freise A.C., Kraft N.J.B., Moberg-Parker J., Sprague J., Kushner D.J., Curd E.E. (2018). Ranacapa: an R package and shiny web app to explore environmental DNA data with exploratory statistics and interactive visualizations. F1000Res..

[5] McMurdie P.J., Holmes S. (2013). Phyloseq: an R package for reproducible interactive analysis and graphics of microbiome census data. PLoS One.

[6] Maniatis T., Fritsch E.F., Sambrook J. (1982).

[7] Chao A. (1984). Non-parametric estimation of the number of classes in a population. Scand. Stat. Theory Appl..

[8] Shannon C.E., Weaver W. (1949).

[9] Simpson E.H. (1949). Measurement of diversity. Nature.

[10] Anderson M.J. (2008). A new method for non-parametric multivariate analysis of variance. Austral. Ecol..

[11] Anders S., Huber W. (2010). Differential expression analysis for sequence count data. Genome Biol.

[12] Kruskal W.H., Wallis W.A. (1952). Use of ranks in one-criterion variance analysis. J. Am. Stat. Assoc..

